# 2224 Determining if intestinal commensal bacteria enhance the frequency of reassortment of an enteric, segmented virus, reovirus

**DOI:** 10.1017/cts.2018.62

**Published:** 2018-11-21

**Authors:** Matthew Lanahan, Andrea Erickson, Julie Pfeiffer

**Affiliations:** 1 Department of Molecular Microbiology, University of Texas Southwestern Medical Center, Dallas, TX, USA

## Abstract

OBJECTIVES/SPECIFIC AIMS: The overall goal is to determine if intestinal commensal bacteria play a role in enteric virus evolution. We will use reovirus, an enteric segmented virus, to investigate specific goals. First, we will determine if specific bacterial species enhance the coinfection frequency of 2 separate strains of reovirus. Second, we will determine if the presence/absence of different bacterial species in the microbiota of mice results in different reovirus reassortment frequencies. Finally, we will discover if reassortant reovirus is present in human populations. METHODS/STUDY POPULATION: My first goal is to determine if specific bacterial species enhance the coinfection frequency of 2 strains of reovirus. In our lab, we have a panel of commensal intestinal bacterial strains, as well as a number of lab adapted bacterial strains. We will use this panel of bacteria to determine if reovirus binds to different species of bacteria using a binding assay involving radiolabeled virus. Additionally, we will determine if specific species of bacteria alter the coinfection frequency through a Flow cytometry based assay. This will involve mixing virus with bacteria, infecting cells in culture, and straining for reovirus proteins for flow cytometry. Our second goal is to determine if specific bacteria promote reassortment of reovirus in a mouse model of infection. To do this, we will use gnotobiotic techniques to create mice harboring different intestinal bacteria populations. Mice will be infected with 2 strains of reovirus, and then feces and organs will be collected. Progeny virus will be subjected to a plaque assay on 2 different types of cells. The first type of cells will be normal cells in culture in which all viable viruses will form plaques. The second will be a cell line that stably expresses siRNAs against specific reovirus segments in which only specific reassortants will form plaques. These 2 plaque assays will be used to quantify the total number of viruses present and the total number of reassortant viruses present. Additionally, SDS-PAGE and RT-PCR will be used to confirm reassortants. Our third goal is to determine if reassortant reovirus is present in infected humans. To do this, I will obtain feces from reovirus-infected children and isolate reovirus. One specific reovirus reassortant is known to propogate in dual-infected mice. I will use the plaque assay technique to determine if this reassortant is also present in humans. To determine if other reassortants are present, I will use RT-PCR and SDS-PAGE. RESULTS/ANTICIPATED RESULTS: Based on previous studies with other enteric viruses, we suspect that specific bacterial species bind reovirus strains with different efficiencies. It is likely that a number of bacterial species will promote coinfection. The bacterial strains that binds both reovirus strains at a high efficiency will likely enhance coinfection by the greatest amount. It is likely that mice harboring different bacterial populations will produce different reovirus reassortment frequencies. We predict that bacteria that enhance reovirus coinfection in vitro should also enhance reovirus reassortment in our mouse model. Therefore, mice specifically lacking bacteria that promote coinfection should have significantly lower amounts of reassortant reovirus. It will be important to control for the overall amount of replication within mice with different microbiotas, as this will affect the basal reassortment frequency. We suspect that reovirus reassortants are present in humans. Work done both in vitro and in mouse models indicates that reassortment happens at high frequencies. Additionally, one specific reassortant commonly propogates in mice due to an enhanced cellular attachment phenotype. Therefore, we predict that this reassortant also commonly emerges after coinfection and reassortment in humans. DISCUSSION/SIGNIFICANCE OF IMPACT: Segmented viruses, such as influenza and rotavirus, are important human pathogens. Viral reassortment poses a unique threat to humans, as it enables new viruses to emerge and cause pandemics or epidemics. However, little is known about what factors promote viral reassortment. This study will provide insight into a novel mechanism of segmented virus evolution.

